# Draft Genome Sequence of *Tetzosporium hominis* VT-49 gen. nov., sp. nov., Isolated from the Dental Decay Plaque of a Patient with Periodontitis

**DOI:** 10.1128/genomeA.01541-17

**Published:** 2018-01-25

**Authors:** George Tetz, Victor Tetz

**Affiliations:** a Human Microbiology Institute, New York, New York, USA; b TGV-Biomed, New York, New York, USA; c Tetz Laboratories, New York, New York, USA

## Abstract

Here, we report the draft genome sequence of *Tetzosporium hominis* VT-49 gen. nov., sp. nov., isolated from the dental plaque of a patient with severe periodontal disease. The draft genome sequence was 2,780,751 bp in length with a 43.3% G+C content. We detected 3,001 genes, which are predicted to encode proteins that regulate both virulence and antibiotic resistance.

## GENOME ANNOUNCEMENT

The novel *Tetzosporium* genus was isolated using previously described combined culture-based and gene-based methods (1–3) from the dental plaque of a patient with severe periodontitis. *T. hominis* VT-49 is a Gram-positive, aerobic, and rod-shaped bacterium.

The 16S rRNA gene of *T. hominis* VT-49 was found to possess ≤96% similarity with different members of the *Planococcaceae* family, namely, *Planococcus* spp., *Paenisporosarcina* spp., and *Sporosarcina* spp. The genome of *T. hominis* VT-49 was sequenced using a HiSeq 2500 platform (GAIIx, Illumina, USA) according to the manufacturer’s instructions, yielding 2,780,751 bp (43.4% G+C content) with a 120-fold coverage. The VT-49 genome sequence was assembled to 393 contigs using SPAdes version 3.5.0 and annotated using the NCBI Prokaryotic Genome Annotation Pipeline (4, 5). The VT-49 genome harbored 66 tRNAs, 15 rRNAs, 4 noncoding RNA operons, and 4,048 protein-coding sequences. Although *in silico* DNA-DNA hybridization (DDH) analysis is required to identify the species within the same order, we performed this analysis to double-check the 16S rRNA gene data. The DDH analysis performed using the genome-to-genome distance calculator algorithm confirmed that the genomes of *T. hominis* VT-49 and *Planococcus* spp., *Paenisporosarcina* spp., and *Sporosarcina* spp. belonged to two different species with a DDH value of less than 25%, which was well below the threshold value of 70% (6, 7).

Analysis of the fatty acids, quinolones, and polar lipids showed that MK-6 (17%), MK-7 (61%), and MK-8 (22%) were the major menaquinones of *T. hominis* VT-49, and the cell wall peptidoglycan belonged to the A4β type containing l-Orn-d-Glu. Simultaneously, *Planococcus* spp. had MK-6 (23.2%), MK-7 (46.4%), and MK-8 (30.3%), and the peptidoglycan type A4α (based on l-Lys-d-Glu); *Paenisporosarcina* spp. had MK-7 and/or MK-8 as the major menaquinones, along with the A4α type of peptidoglycan (l-Lys-d-Asp); and *Sporosarcina* spp. had MK-7 (>90%) with MK-6 (~2%) and the peptidoglycan type A4α l-Lys-Gly-d-Glu (8–10). Based on these characteristics, strain VT-49 was assigned to the novel genus and species* T. hominis*.

In the VT-49 genome, we identified the presence of protein-coding genes that confer resistance to antibiotics, such as the lactam utilization protein LamB, an organic hydroperoxide resistance protein, as well as multidrug-resistant transporters of the ATP-binding cassette (ABC) family, the multidrug and toxic compound extrusion (MATE) family, and the major facilitator superfamily (MFS). Virulence factors, such as hemolysin D, metalloproteases, peptidases, deoxyribonucleases, ribonucleases, flagellar components, and adhesins, were identified in the VT-49 genome. Moreover, we identified the virulence factors that were typical to the collagenase-like protease and amine oxidase of other periodontal pathogens (11). Follow-up studies on *T. hominis* VT-49 and bacteriophages associated with this microorganism would enable us to understand explicitly its implications in human pathologies and its possible role in periodontal pathologies (12, 13).

### Accession number(s).

This complete genome sequence has been deposited in GenBank under the accession no. NOKQ00000000.

## References

[1] Tetz G, Tetz V 2017 Introducing the sporobiota and sporobiome. Gut Pathog 9:38. doi:10.1186/s13099-017-0187-8. 28680484 PMC5493122

[2] Tetz G, Tetz V, Vecherkovskaya M 2016 Genomic characterization and assessment of the virulence and antibiotic resistance of the novel species *Paenibacillus* sp. strain VT-400, a potentially pathogenic bacterium in the oral cavity of patients with hematological malignancies. Gut Pathog 8:6. doi:10.1186/s13099-016-0089-1. 26900405 PMC4761199

[3] Tetz G, Tetz V 2015 Complete genome sequence of *Bacilli bacterium* strain VT-13-104 isolated from the intestine of a patient with duodenal cancer. Genome Announc 3(4):e00705-15. doi:10.1128/genomeA.00705-15. 26139715 PMC4490844

[4] Bankevich A, Nurk S, Antipov D, Gurevich AA, Dvorkin M, Kulikov AS, Lesin VM, Nikolenko SI, Pham S, Prjibelski AD, Pyshkin AV, Sirotkin AV, Vyahhi N, Tesler G, Alekseyev MA, Pevzner PA 2012 SPAdes: a new genome assembly algorithm and its applications to single-cell sequencing. J Comput Biol 19:455–477. doi:10.1089/cmb.2012.0021. 22506599 PMC3342519

[5] Tatusova T, DiCuccio M, Badretdin A, Chetvernin V, Ciufo S, Li W 2013 Prokaryotic genome annotation pipeline. *In* Beck J, Benson D, Coleman J, Hoeppner M, Johnson M, Maglott D, Mizrachi I, Morris R, Ostell J, Pruitt K, Rubinstein W, Sayers E, Sirotkin K, Tatusova T (ed), The NCBI handbook, 2nd ed. National Center for Biotechnology Information, Bethesda, MD.

[6] Auch AF, von Jan M, Klenk HP, Göker M 2010 Digital DNA-DNA hybridization for microbial species delineation by means of genome-to-genome sequence comparison. Stand Genomic Sci 2:117–134. doi:10.4056/sigs.531120. 21304684 PMC3035253

[7] Chun J, Rainey FA 2014 Integrating genomics into the taxonomy and systematics of the *Bacteria* and *Archaea*. Int J Syst Evol Microbiol 64:316–324. doi:10.1099/ijs.0.054171-0. 24505069

[8] Reddy GSN, Manasa BP, Singh SK, Shivaji S 2013 *Paenisporosarcinaindica* sp. nov., a psychrophilic bacterium from a glacier, and reclassification of *Sporosarcina antarctica* Yu et al., 2008 as *Paenisporosarcina antarctica* comb. nov. and emended description of the genus *Paenisporosarcina*. Int J Syst Evol Microbiol 63:2927–2933. doi:10.1099/ijs.0.047514-0. 23355696

[9] Kaur I, Das AP, Acharya M, Klenk HP, Sree A, Mayilraj S 2012 *Planococcus plakortidis* sp. nov., isolated from the marine sponge *Plakortis simplex* (Schulze). Int J Syst Evol Microbiol 62:883–889. doi:10.1099/ijs.0.029967-0. 21642486

[10] Reddy GSN, Matsumoto GI, Shivaji S 2003 *Sporosarcina macmurdoensis* sp. nov., from a cyanobacterial mat sample from a pond in the McMurdo Dry Valleys, Antarctica. Int J Syst Evol Microbiol 53:1363–1367. doi:10.1099/ijs.0.02628-0. 13130019

[11] Loesche WJ 1996 Microbiology of dental decay and periodontal disease. *In* Baron S (ed), Medical microbiology, 4th ed. University of Texas Medical Branch at Galveston, Galveston, TX. 21413316

[12] Tetz GV, Ruggles KV, Zhou H, Heguy A, Tsirigos A, Tetz V 2017 Bacteriophages as potential new mammalian pathogens. Sci Rep 7:7043. doi:10.1038/s41598-017-07278-6. 28765534 PMC5539208

[13] Tetz G, Tetz V 2017 Prion-like domains in phagobiota. Front Microbiol 8:2239. doi:10.3389/fmicb.2017.02239. 29187840 PMC5694896

